# Botulinum toxin related research in maxillofacial plastic and reconstructive surgery

**DOI:** 10.1186/s40902-016-0080-2

**Published:** 2016-09-05

**Authors:** Tae-Geon Kwon

**Affiliations:** Department of Oral and Maxillofacial Surgery, School of Dentistry, Kyungpook National University, 2177 Dalgubeoldaero, Jung Gu, Daegu, 41940 South Korea

Botulism caused by food poisoning was characterized by mydriasis and skeletal muscle paralysis, which was first described by Justinus Kerner in 1820 [1]. The cause of botulism was botulinum neurotoxin produced by anaerobic, spore-forming bacteria of the genus *Clostridium* [2]. Botulinum toxin (BTX) became to be the first medically applied toxin. The first clinical use of BTX was reported concerning the treatment of strabismus in ophthalmologic field in 1980 [3]. Nine years later, the Food and Drug Administration (FDA) approved the clinical application of BTX for adult strabismus and blepharospasm [4]. BTX inhibits acetylcholine exocytosis at neuromuscular junction of the preganglionic sympathetic/parasympathetic nerve fibers and postganglionic parasympathetic nerves [5].

BTX is clinically administrated to treat various therapeutic indications: strabismus, migraine, bladder dystonia, hyperhidrosis, cervical dystonia, upper limb spasticity, voice abnormality, chronic pain management, and cerebral palsy [6]. Recently, the most popular indication in public is the control of facial wrinkle [7]. In oral and maxillofacial surgery field, BTX injection is applied not only for cosmetic purposes such as glabella line correction, platysma band correction, or gummy smile but also for therapeutic indications such as masseteric or temporalis muscle hypertrophy [8–10], temporomandibular joint disorders [9, 11], salivary gland secretory disorders (including sialorrhea, Frey syndrome) [12], hypertrophic scars [13], facial pains [14], and facial paralysis [15, 16]. Especially in dental field, it had been reported that BTX improved painful symptoms as high as 90 % of temporomandibular joint disorders related to masticatory muscles [17]. In cosmetic purpose in dental field, excessive gingival exposure during smile or asymmetric smile can be corrected by the BTX injection into peri-oral muscles [18, 19]. Therefore, even though BTX treatment had been historically dermatologists and neurologists’ jurisdiction, it now became the dentists’ jurisdiction because the training and scientific knowledge covers the entire head and neck region [19].

However, there are a number of complications associated with the BTX injection especially related with the accidental overdose. According to the previous report, there are various local and systemic side effects after BTX injection. Pain, edema, headache, and bruising would be the common loco-regional side effects, and nausea, fatigue, headache, facial pain, flu-like symptoms, anxiety, and itching can appear as systemic side effects after BTX administration [1, 20]. In 2005, it was reported that the adverse event reported to the FDA (Dec 1989~May 2003) after therapeutic and cosmetic use of BTX was 1437 cases. Among these, 217 serious adverse events were reported including 28 reported deaths; respiratory arrest (*n* = 6), myocardial infarction (*n* = 5), cerebrovascular accident (*n* = 3), pulmonary embolism (*n* = 2), and others (*n* = 3) [21]. All of them were related to therapeutic application rather than cosmetic purpose of the BTX.

Even though there are numerous publications reporting successful outcomes after BTX application, there are only a few scientific reports with high level of scientific evidence. Because the clinical research had not been carried out with randomized, controlled, blinded settings, more clinical and experimental research should be encouraged. Clinical research need to be carried out more tightly under controlled condition with a prospective, randomized design rather than demonstration of successful case series. At the same time, to overcome fundamental limitations of the BTX, the basic research is needed to advance and improve the clinical application of BTX.
